# The role of atmospheric internal variability on the prediction skill of interannual North Pacific sea-surface temperatures

**DOI:** 10.1007/s00704-017-2169-7

**Published:** 2017-06-06

**Authors:** Balachandrudu Narapusetty

**Affiliations:** 1 Atmospheric, Oceanic, and Earth Sciences, George Mason University, Fairfax, VA USA; 2 Innovim/Climate Prediction Center/NCEP/NWS, College Park, MD USA; 3 NOAA Center for Weather and Climate Prediction, 5830 University Research Court, College Park, MD 20740 USA

## Abstract

The sensitivity of the sea-surface temperature (SST) prediction skill to the atmospheric internal variability (weather noise) in the North Pacific (20^∘^–60^∘^N;120^∘^E–80^∘^W) on decadal timescales is examined using state-of-the-art Climate Forecasting System model version 2 (CFS) and a variation of CFS in an Interactive Ensemble approach (CFSIE), wherein six copies of atmospheric components with different perturbed initial states of CFS are coupled with the same ocean model by exchanging heat, momentum and fresh water fluxes dynamically at the air-sea interface throughout the model integrations. The CFSIE experiments are designed to reduce weather noise and using a few ten-year long forecasts this study shows that reduction in weather noise leads to lower SST forecast skill. To understand the pathways that cause the reduced SST prediction skill, two twenty-year long forecasts produced with CFS and CFSIE for 1980-2000 are analyzed for the ocean subsurface characteristics that influence SST due to the reduction in weather noise in the North Pacific. The heat budget analysis in the oceanic mixed layer across the North Pacific reveals that weather noise significantly impacts the heat transport in the oceanic mixed layer. In the CFSIE forecasts, the reduced weather noise leads to increased variations in heat content due to shallower mixed layer, diminished heat storage and enhanced horizontal heat advection. The enhancement of the heat advection spans from the active Kuroshio regions of the east coast of Japan to the west coast of continental United States and significantly diffuses the basin-wide SST anomaly (SSTA) contrasts and leads to reduction in the SST prediction skill in decadal forecasts.

## Introduction

The variability in the SST on interannual time-scales in the North Pacific Region (NPR; 20^∘^-60^∘^N -120^∘^E-100^∘^W) is an important aspect in understanding the Pacific decadal variability (Mantua et al. 1997; Power et al. 1999; Deser et al. 2004). The SST variability in the NPR is generally characterized by strong atmosphere-ocean variations (Trenberth and Hurrell 1994) and this variability is best described as a combination of multiple physical modes (Deser et al. 2010).

Several mechanisms are proposed to explain the variability of SST in the NPR. These mechanisms are broadly generalized into three categories: (1) stochastic forcing of weather noise, (2) remote forcing of El Niño Southern Oscillation (ENSO) through atmospheric bridge phenomenon, and (3) subsurface ocean heat transport from Kuroshio and its extension. The view of the first category is that due to the higher heat capacity of oceans in the earth-climate system, stochastic forcing by weather noise acts to redden the low-frequency Ocean SST variability spectra (Hasselmann 1976; Frankignoul and Hasselmann 1977; Barsugli and Battisti 1998). The second category is based on the statistical correlations that lead to hypothesize that the variability in the North Pacific SSTs are dominated by air-sea interactions that are remotely forced through atmosphere by ENSO SST anomalies (Alexander et al. 2002). The third category stems from the simple conceptual models in association with analyzing in-situ datasets (Deser et al. 1996; Saravanan and McWilliams 1998; Qiu 2003) as recent advances in the satellite imagery and ocean data assimilation that allow to examine the volumetric heat transport from Kuroshio and Ryuku current streams (Andres et al. 2009); these subsurface heat transport processes are shown to influence the North Pacific SSTs on decadal time scales.

There are also few studies that examine the SST variability in the NPR as a combination of these categories. For instance, NPR variability is argued to be a combination of stochastic forcing due to the weather noise and the ENSO forcing through atmospheric bridge (Newman et al. 2003). SST variability in the NPR is also proposed as a combination of the weather noise forcing and the SST reemergence mechanism due to the seasonal variations in the mixed layer depths (Deser et al. 2003). The impact of subsurface heat transport variations on SST variability due to the wind stress variability and the weather noise forcing were also examined in the previous studies. A study by Deser et al. (1996) showed that a lead-lag correlation between mean curl of the wind stress and subsurface thermal anomalies in the NPR is established through the ventilated thermocline mechanism (Luyten et al. 1983). Though not directly linked to SST variability in the NPR, the interannual heat content variations in the Kuroshio and its extension were well captured by forcing a simplified ocean model by predominantly Rossby wave driven atmospheric momentum (Kawabe 2001). However, the pathways that link the subsurface heat content and the SST in the NPR could be well examined with coupled general circulation model (CGCM) experiments.

This study is motivated by advancements in the coupled modeling that allow to realistically separate impact of the atmospheric internal dynamics (weather noise) on North Pacific SST. The primary goal is to understand the pathways that influence the variability in the North Pacific SST in the absence of weather noise at the ocean-atmosphere interface in a CGCM. To achieve this a CGCM simulation should be compared against the exact same CGCM counterpart that has no weather noise. In this study, the impact of weather noise on the North Pacific SST variability is analyzed by comparing a twenty-year long forecast produced by state-of-the-art Climate Forecasting System version 2 (CFS; Saha et al. (2012)) against another identical forecast produced by the same model but with reduced weather noise.

Interactive Ensemble method (Kirtman and Shukla 2002) is employed to reduce the weather noise fluxes of heat, momentum and fresh water at the ocean-atmosphere interface in the CFS forecasts. This variant of CFS in Interactive Ensemble framework (CFSIE) produces a modified version of CFS by coupling the ocean component with the averaged state of fluxes from several atmospheric components. To completely eliminate the weather noise, fluxes produced by infinitely large number of atmospheric components at air-sea interface shall have to be coupled with the ocean component at an infinitesimal time intervals during the model integrations. However, this study utilizes six realizations of atmospheric components to interact with one ocean component at a model integration time step of every 30 mins as the previous studies show that coupling the mean of six atmospheric realizations with ocean component at half hour integrations can significantly reduce the weather noise (Kirtman et al. 2005; Stan and Kirtman 2008). This way of reducing weather noise was shown to influence the low-frequency SST variability in CGCMs (Yeh and Kirtman 2004; Schneider and Fan 2007).

The manuscript is organized as follows. Section 2 contains the numerical experiments that produced the 20-year long Climate Forecasting System forecast and a similar forecast with reduced weather noise and the decadal forecast data that is used in deducing the SSTA prediction skill in CFS and CFSIE forecasts. The analysis of the CFS and CFSIE experiments along with discussions are presented in Section 3 and conclusions of this study are summarized in Section 4.

## Description of numerical experiments

Several ten-year long forecasts are produced with CFS and CFSIE to examine the prediction skill in the decadal forecasts are detailed below. Two twenty-year long forecasts with CFS and CFSIE are also performed to understand the differences of underlying processes in the absence of weather noise. All the forecasts produced in this study are initialized only in the beginning and no initialization or data assimilation is performed during the course of the model integration.

### Twenty-year long forecasts

The twenty-year long forecast produced from the state-of-the-art coupled global forecast model CFS is compared against similar forecast produced by CFSIE configuration (Kirtman and Shukla (2002)). A brief discussion on the atmosphere and ocean model components used in the CFS is documented by Narapusetty et al. (2012).

In the Interactive Ensemble configuration, the momentum, heat and fresh water fluxes that atmospheric component exchanges with ocean component are controlled in a way that the higher frequencies in these fluxes are reduced at the ocean-atmosphere interface. This is achieved by concurrently coupling the mean of the fluxes simulated by several atmospheric components with a single ocean component that in turn forces each atmospheric component with the same SST. This setup leads to a reduction in the atmospheric internal variability in the coupled system. For a more detailed discussion on how the Interactive Ensemble approach reduces the weather noise in a CGCM, refer to Kirtman et al. (2005).

The twenty-year long CFS and CFSIE forecasts are produced from 1 November 1980 to 31 October 2000. The atmosphere and the ocean initial conditions for the forecasts are derived from CFS reanalysis products (available online at http://cfs.ncep.noaa.gov/cfsr/). The initial conditions needed for the six copies of the atmospheric components in CFSIE integration are obtained from the CFS reanalysis data separated by 6 hours starting from 00Z of 1 November 1980.

### Ten-year long forecasts

**Fig. 1:**
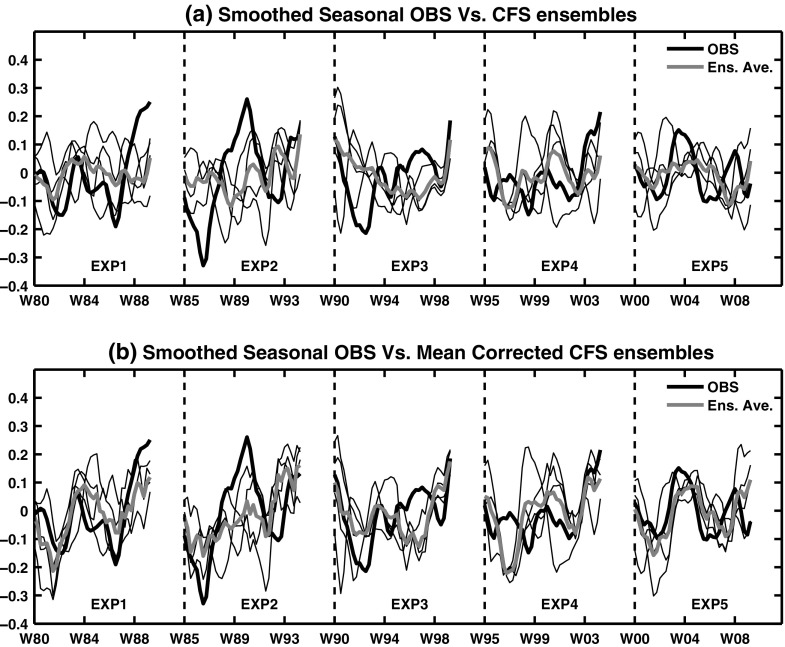
Time evolution of the PAA index shown in thick black lines for (**a**) CFSf seasonal forecasts, and (**b**) same forecasts, but by applying a simple bias correction. In both the panels, the thick grey line depicts ensemble mean, and the thick black line corresponds to observations. X-axis shows all the ten initialized years with each initialized year separate with vertical dashed line

Two sets of few 10-year forecasts from CFS and CFSIE (referred as CFSf and IEf hereafter) are produced in this study to understand the SST prediction skill in the NPR. The CFSf are a set of monthly forecasts that follow the guidelines set forth by Coupled Model Intercomparison Project phase 5 (CMIP5; http://pcmdi3.llnl.gov/esgcet/), and are run in the 20th century historical external forcing. The CFSf are initialized with NEMOVAR-COMBINE ocean reanalysis data (Saha et al. 2012) for the ocean component of the model and NCEP CFS Reanalysis and Reforecast (CFSRR) data for the atmospheric component. Each forecast in the CFSf consists of four ensemble members, and each ensemble member is obtained by running the CFS for ten years initialized in the month of November of 1980, 1985, 1990, 1995, and 2000. For each initialized forecast, the seasonal anomaly of individual forecast is estimated by subtracting the annual cycle (Narapusetty et al. 2009) of the mean- ensemble forecast and average the monthly anomalies across seasons. These seasonal anomalies are smoothed to retain the low-frequency variability as described by Trenberth and Hurrell (1994). Hereafter, the smoothed seasonal anomalies are referred as seasonal forecasts for brevity. In addition, these seasonal forecasts are also bias corrected based on a simple mean correction as discussed by Narapusetty et al. (2014), and are shown separately for Pacific area average (PAA) index that is constructed as the average of SST anomalies over 20^∘^-60^∘^N and 120^∘^E-100^∘^W (Fig. 1). The mean bias correction has reduced the model-induced drift in the ensembles and has improved the internal variability in some forecast experiments (e.g., EXP5 in Fig. 1).

**Fig. 2:**
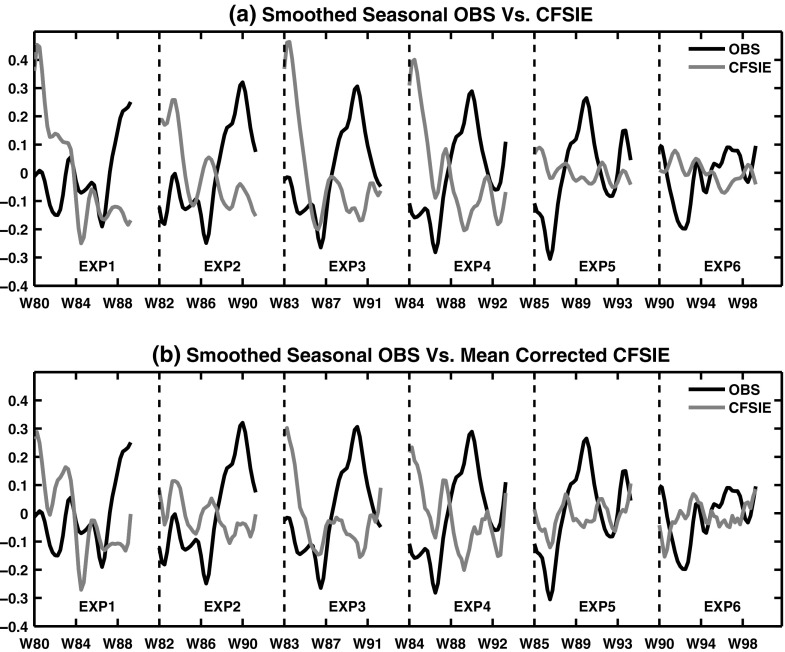
Same as Fig. 1, except for IEf

The IEf are a single member 10-year forecasts starting from the month of November in the years 1980, 1982, 1983 and 1984 (Fig. 2). The 1985-1995 and 1990-2000 forecasts as shown in the Fig. 2 are extracted from the 20-year long CFSIE long forecast runs. The oceanic and atmospheric components are initialized with NCEP CFSRR data. Seasonal anomalies and smoothed seasonal forecasts are estimated exactly similar as in the case of CFSf ensembles explained above. However, the bias correction to the seasonal forecasts was performed with a 20-year long CFSIE forecast obtained for the period of 1980 to 2000 as opposed to using the observed data for correcting the biases as in the CFSf as the model drift corrected with observation could introduce artificial trends. Note that the bias corrected seasonal forecasts of PAA index in IEf show relatively closer proximity to the longer CFSIE forecast (Fig. 2).

## Results and discussion

### Prediction skill of North Pacific SSTs

**Fig. 3:**
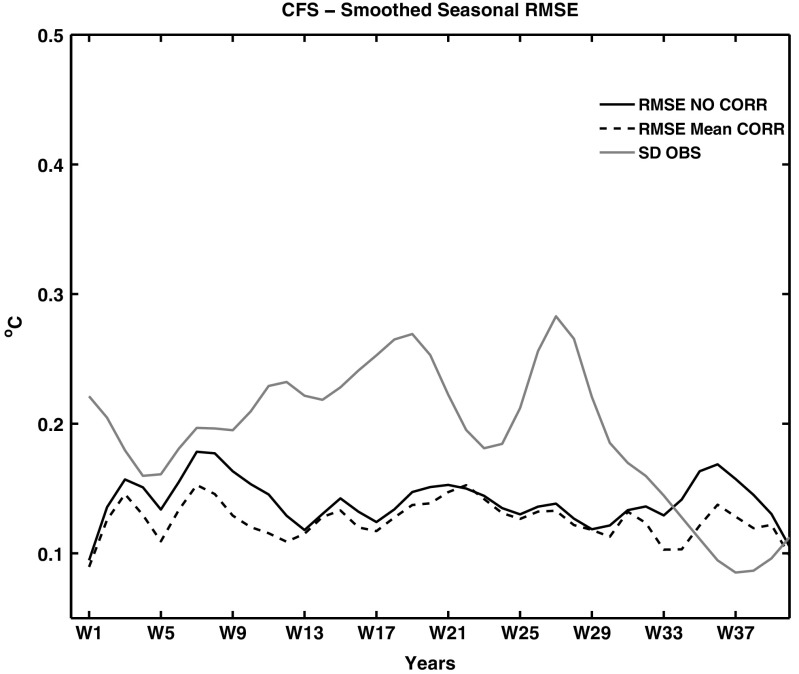
Root mean square error of the PAA index (^∘^C) as a function of lead time for CFSf smoothed seasonal forecasts in *black line* and mean corrected forecasts in *dashed line*. The *grey line* depicts the standard deviation of the observations. X-axis depicts the lead seasons

**Fig. 4:**
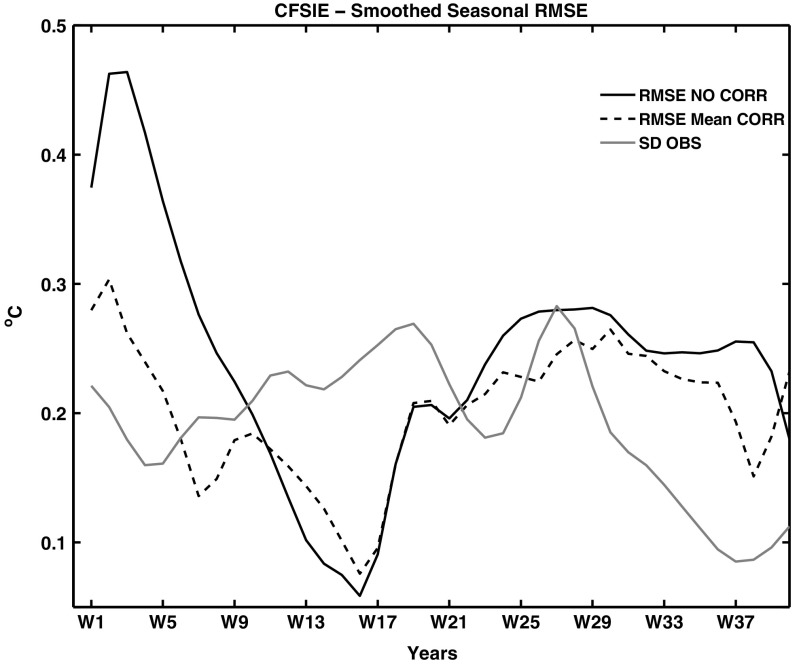
Same as Fig. 3, but for seasonal IEf forecasts

Figures 3 and 4 show the root mean square errors (RMSEs) of the PAA index for the 10-year seasonal CFSf and IEf forecasts as a function of lead season. The RMSEs, calculated as a function of lead season are compared against standard deviation of observations (SDO) to understand the deterministic prediction skill of the forecasts. Lower values of RMSEs compared to the corresponding SDO indicate the existence of prediction skill in the forecast. For CFSf forecasts, the RMSEs of the PAA index are well below the SDO and the employed bias correction technique further lowered the RMSEs (Fig. 3). In the case of IEf forecasts, the RMSEs are lower than the SDO only from lead seasons 10 to 20. The bias corrected forecasts reduce the RMSEs during the initial 10 seasons and this results in the RMSEs being lower than the SDO from 6 to 20 seasons. The RMSEs of seasonal forecasts suggest that the CFSf forecasts have prediction skill up to 35 seasons, where as the IE forecasts are skillful only up to 20 seasons.

### Relationship between Kuroshio current on North Pacific SSTs

In this section, the influence of heat transport by Kuroshio and its extension on north Pacific SSTs is explored to understand the lower prediction skill that is detected in the IEf. The 20-year long integrations for CFS and CFSIE experiments as explained in Section 2a are used to examine the physical processes that lead to the change in the prediction skills between CFSf and IEf forecasts.

**Fig. 5:**
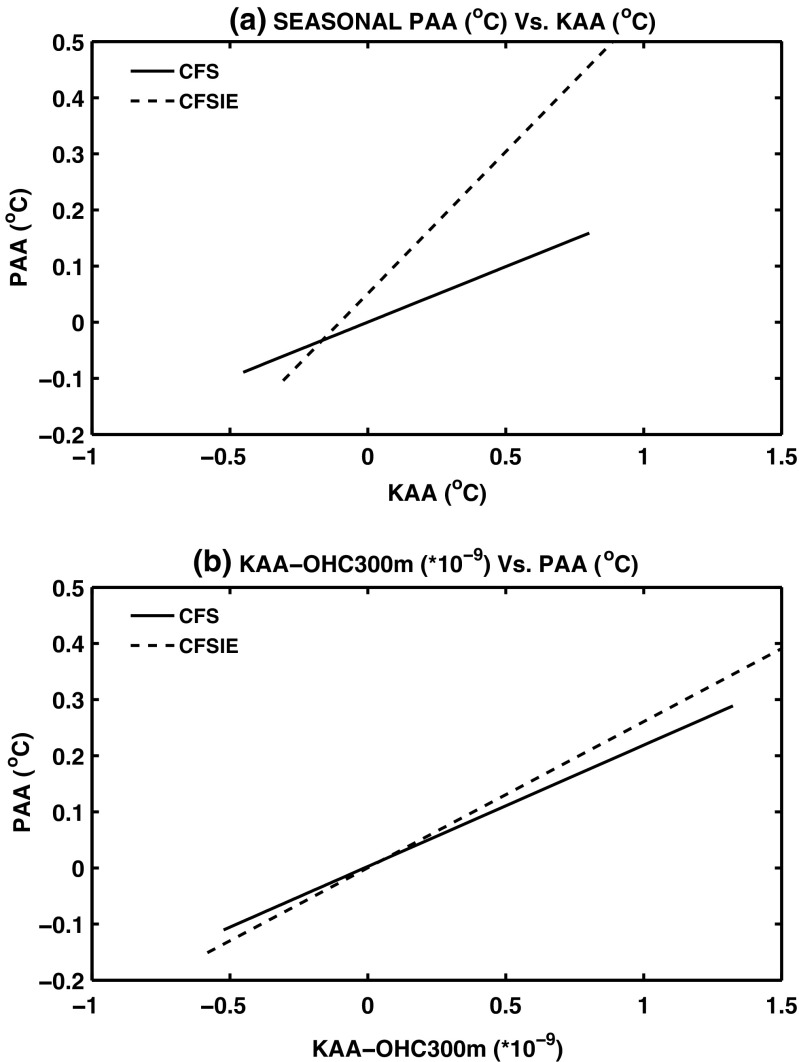
Regression coefficient as estimated between (**a**) KAA and PAA (^∘^C/^∘^C) and (**b**) 300m accumulated ocean heat content in Kuroshio average and PAA. In both the panels solid line indicates CFS and dashed line represents IE

Linear regression analysis suggests that, in both CFS and CFSIE the changes in the PAA index are explained by the SSTA averaged in Kuroshio region (20^∘^-35^∘^N; referred as KAA hereafter). However, in the CFSIE the contribution from the KAA index on PAA index is larger (Fig. 5a), suggesting a stronger linkage between Kuroshio region with the NPR. This linkage is further strengthened by the fact that the ocean heat content accumulated in the top 300 m (OHC300 hereafter) in the KAA index contributes more to SSTA variability in the PAA index than in CFS (Fig. 5b).

**Fig. 6:**
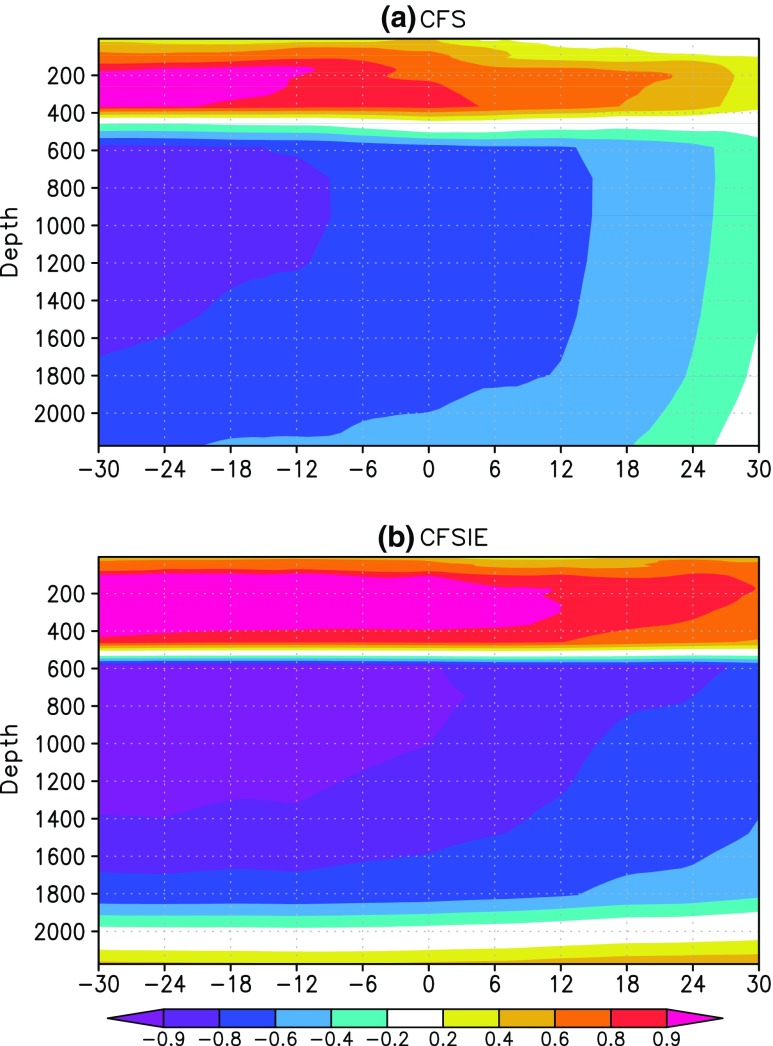
Lead-lag correlation between OHC300 and ocean temperature anomalies in the PAA index of all depths (**a**) CFS and (**b**) CFSIE. X-axis shows the months. Negative values on X-axis refer to OHC300 leading ocean temperatures and vice versa

The difference in CFS and CFSIE SSTA in the PAA index in terms of their dependance on the OHC300 is also seen on the ocean temperatures for the depths up to 300m. Lead-lag correlations between OHC300 and ocean temperature anomalies indicate that in the CFSIE, OHC300 leads SSTA in the PAA index much strongly than in the CFS (Fig. 6). The regression analysis (Fig. 5) as well as correlation between OHC300 and SSTA suggest that the ocean heat transport plays an important role in establishing the relation between Kuroshio and the rest of the North Pacific region.

To understand the relation between weather noise and ocean heat transport in the North Pacific, heat budget is analyzed in the oceanic mixed layer in the CFS and the CFSIE experiments. The heat budget analysis is restricted for the Boreal cold season months from October to February as during these months the oceanic mixed layer response to the atmospheric forcing is high in this region (Frankignoul 1985; Cayan 1992). Heat budget in the mixed layer is analyzed using the following equation as discussed in detail by (Tomita et al. 2002) and used on various CGCM outputs by Kang et al. (2010) and Yim et al. (2012):

1 $$\begin{array}{@{}rcl@{}} {\int\limits_{0}^{H}}{\!{\int\limits_{t_{i}}^{t_{l}} \!\mathrm{T}_{t} \, \mathrm{d} t}} \, \mathrm{d} z &=& \int\limits_{t_{i}}^{t_{l}} \!{\frac{Q^{n}}{{\rho}c_{p}}} \, \mathrm{d} t + {\int\limits_{0}^{H}}\!\int\limits_{t_{i}}^{t_{l}} \left\{\!{-\overrightarrow{\mathbb{V}}\!\!\odot\!\!\overrightarrow\nabla{\mathrm{T}}}\right.\\ &&+\left.\overrightarrow\nabla\!\!\odot\!\!\left[\overrightarrow\nabla{\nu_{h}\!\mathrm{T}}\right]\right\} \, \mathrm{d} t \, \mathrm{d} z\\ &&+ \int\limits_{t_{i}}^{t_{l}}\left\{-{\textsl{w}}{\mathrm{T}_{z}} + \left( \nu_{v}\!\mathrm{T}_{z}\right)_{z}\right\}|_{}^{z=H} \mathrm{d} t, \end{array} $$

where T_*t*_ and T_*z*_ are the derivatives of ocean temperatures in time and vertical direction, *Q* ^*n*^ is the net heat flux at the sea surface, *ρ* and *c*_*p*_ are density and specific heat at constant pressure for seawater, $\nu _{_{\!\!h}}$ and $\nu _{_{\!\!v}}$ represent horizontal and vertical eddy diffusivities, respectively. The time integration is performed from October through February in each year and this is shown with *t*_*i*_ and *t*_*l*_, respectively. The integration parameter ‘H’ indicates the depth of mixed layer at the end of winter, that is in February. The mixed layer depth (MXLD) in each month is estimated by interpolating the ocean depths, between which, the ocean temperature differs from the SST by 0.5^∘^C (Levitus 1982). $\overrightarrow {\mathbb {V}}$ represents the horizontal velocity ($\hat {i} \, \mathrm {u}+\hat {j} \, \mathrm {v}$) that includes both geostrophic and Ekman components.

The LHS of Eq. 1 explains the variation of heat in the mixed layer (referred to as VH hereafter), second term in the RHS explains the heat fluxes by advection and diffusion, and the third term includes the advective and diffusive fluxes at the interface z=H that are accounted for the change in the mixed layer depths from October to February. The second and third terms together in the RHS explain the heat transfer due to advective and diffusive fluxes (referred to as HT hereafter). Therefore, from the heat budget equation (Eq. 1), ocean heat transport in the mixed layer could be estimated as HT = VH −Q^∗^, where Q^∗^ is the first term in the RHS of Eq. 1 that represents the average heat flux at the sea surface.

**Fig. 7:**
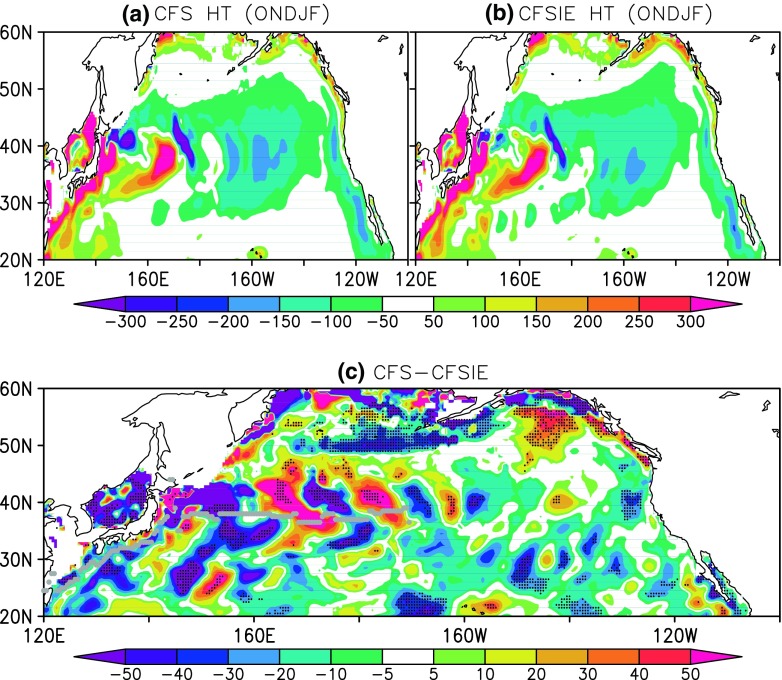
Heat transfer in the mixed layer as estimated for (**a**) CFS, (**b**) CFSIE, and (**c**) difference between CFS and CFSIE. Stippling denotes 5% significant differences and the grey dotted line represents the path of Kuroshio current and its extension

**Fig. 8:**
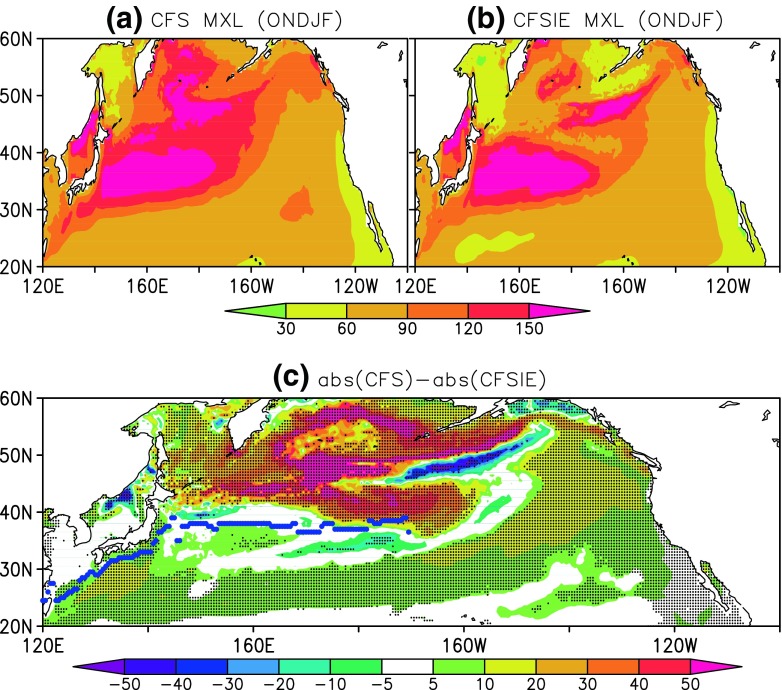
MXLD (m) as estimated in (**a**) CFS, (**b**) CFSIE, and (**c**) difference between CFS and CFSIE. Stippling denotes 5% significant differences and the blue dotted line represents the path of Kuroshio current and its extension

The estimated HT is relatively stronger in the CFSIE forecast in the Kuroshio region, in the North Pacific above 50^∘^N, and most part of the east of dateline (Fig. 7). The HT in the CFS forecast is slightly stronger in some areas west of dateline and around 40^∘^N, however, the 5% statistically significant differences show that HT in the CFSIE is stronger predominantly. The higher HT values in the CFSIE could be traced back to the mixed layer variability between CFS and CFSIE. The difference in the MXLD between CFS and CFSIE reveals that the deeper MXLD in the CFS (Fig. 8) prompts for the higher heat storage requirement, therefore has constrained the horizontal heat advection. The occurrence of shallower MXLD in the CFSIE is understood as a straightforward consequence of reduced atmospheric internal variability due to the reduced variance of the momentum flux in CFSIE at the air-sea interface. The Interactive Ensemble approach employed in CFSIE to diminish the weather noise reduced the momentum flux around 6 times compared to that of the CFS (not shown). In the CFS, the stronger variance in the momentum flux around 40^∘^N coincides with the deeper mixed layers in that region and reduction in the HT.

**Fig. 9:**
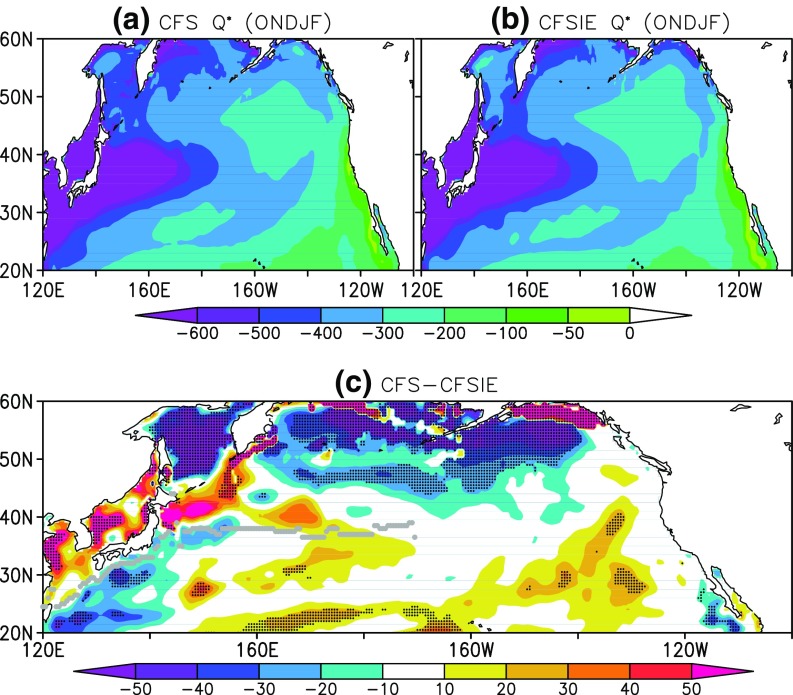
Surface heat flux (Q^∗^ in^∘^C-m) as calculated in (**a**) CFS, (**b**) CFSIE, and (**c**) difference between CFS and CFSIE. Stippling denotes 5% significant differences and the grey dotted line represents the path of Kuroshio current and its extension

Throughout the NPR, the CFS forecast is marked with deeper mixed layers and this higher MXLD in CFS also prompts higher surface heat flux exchange at the ocean-atmosphere interface. The CFS forecast is marked with higher surface heat flux exchange between the ocean and the atmosphere in the North Pacific above 40^∘^N and in the Kuroshio region; however, in the Kuroshio extension region and below 40^∘^N the differences between CFS and CFSIE are small (Fig. 9). By the design of interactive ensemble approach, the heat flux at the air-sea interface should generally be lower compared to CFS forecast; however, in the vicinity of 50^∘^N and beyond the CFSIE produces higher higher heat flux. This is due to the fact that the IE averaging is not performed at the air-sea interface whenever sea-ice is formed. The IE strategy over freezing ocean is known to produce spurious changes in the sea-ice (Kirtman et al. 2011) and to prevent such large variations, only the fluxes produced by the first atmospheric component is coupled to the ocean component around the freezing temperatures.

**Fig. 10:**
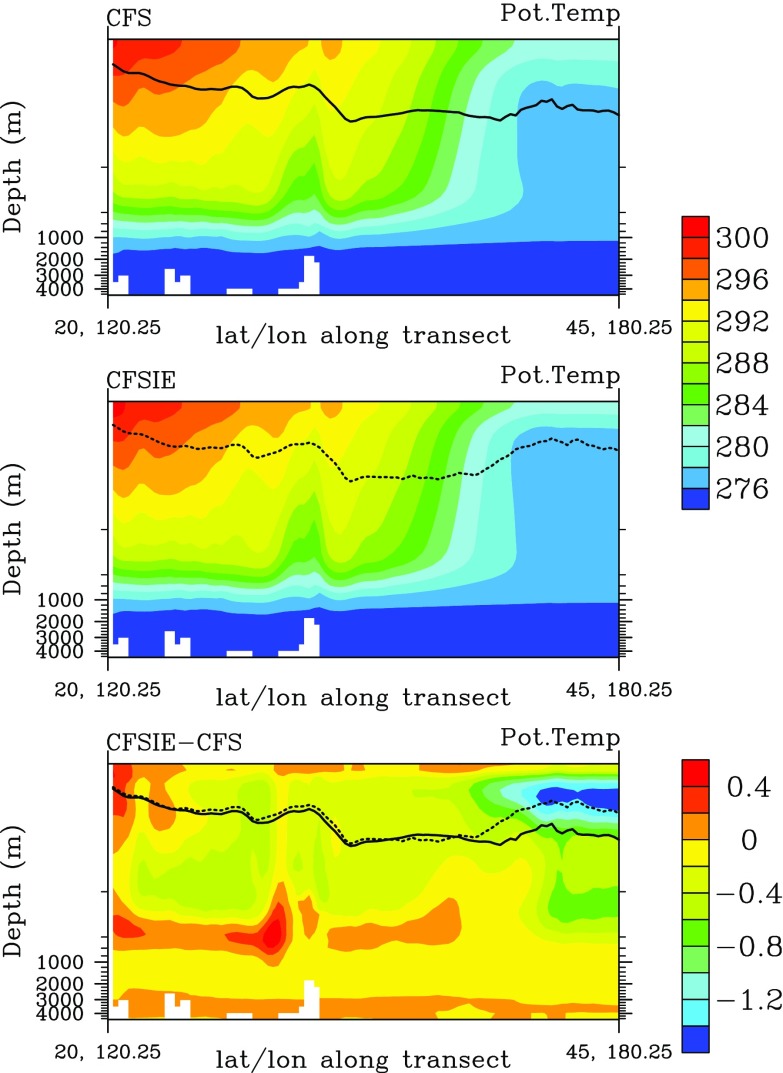
Depth-cross section of SST along the transect 20^∘^N;120.25^∘^E-45^∘^N;180.25^∘^E for (**a**) CFS, (**b**) CFSIE and (**c**) difference between CFSIE and CFS. The solid and dashed black lines show the MXLD for CFS and CFSIE, respectively

The higher HT detected in the CFSIE forecast is well correlated with the PAA index. The regression of standard deviation of PAA index (SD_PAA) on standard deviation of HT (SD_HT) shows that in the CFSIE, HT contributes more to the variance of PAA index (not shown). The strong spatial contrast of the regression parameter in the NPR indicates that HT acts to diffuse the variance of PAA index. The higher HT in conjunction with the lower MXLD in the CFSIE forecast prompts to diffuse stronger SST contrasts that are specific to decadal SST forecasts in the North Pacific region that were found in the CFS forecasts. The depth cross-section of averaged SST along the transect that connects the locations 20^∘^N;120.25^∘^E and 45^∘^N;180.25^∘^E with a greater circle reveals that the SST and its gradient in the CFSIE forecast is indeed weaker than in the CFSIE well below the mixed layer during the winter seasons (Fig. 10).

## Conclusions

The motivation for this study stems from the reduced prediction skill in North Pacific region (20^∘^-60^∘^N and 120^∘^E-80^∘^W, referred as NPR) SSTA forecasts as produced by CMIP5-style decadal forecasts with state-of-the-art global climate forecasting system model (CFS) in an Interactive Ensemble set-up (CFSIE), which reduces atmospheric internal variability. The main goal of this study is to examine and understand the pathways that prompted the reduced prediction skill in the CFSIE forecasts in comparison with the regular CFS forecasts. Two 20-year long forecasts produced with CFS and CFSIE for 1980-2000 are compared for the subsurface characteristics that influence SST in the North Pacific Region. The detailed heat budget analysis in the oceanic mixed layer of the CFS and CFSIE experiments reveals that the heat transport in the oceanic mixed layer is driven significantly by the atmospheric internal variability (weather noise). In the CFSIE, the reduction in the weather noise leads to shallower mixed layer depths and the increase in the heat content variations associated with lower heat flux result in diminished heat content storage and enhanced horizontal heat advection throughout the basin. Due to the deeper mixed layer in CFS, the available heat flux channels to raise the temperature in the the mixed layer and this diminishes the heat available for the basin-wide horizontal transport.

The enhanced HT in the CFSIE is basin-wide in the spatial extent and spans from the active Kuroshio regions of east coast of Japan to the west coast of continental United States. This enhanced horizontal advection of heat in the oceanic mixed layer acts in a way to diffuse the SSTA contrasts and leads to reduced skills in decadal forecasts.

## References

[CR1] Alexander M, Blade I, Newman M, Lanzante JR, Lau NC, Scott JD (2002). The atmosperhic bridge: The influence of ENSO teleconnections on air-sea interaction over the global oceans. J Clim.

[CR2] Andres M, Park J-E, Wimbush M, Zhu X-H, Nakamura H, Kim K, Chang K-I (2009) Manifestation of the Pacific decadal oscillation in the Kuroshio. Geophys Res Lett, 36 (L16602)

[CR3] Barsugli J, Battisti DS (1998). The basic effects of atmosphere-ocean thermal coupling on midlatitude variability. J Atmos Sci.

[CR4] Cayan DR (1992). Latent and sensible heat flux anoMalies over the northern oceans: Driving the sea surface temperature. J Phys Oceanogr.

[CR5] Deser C, Alexander M, Xie SP, Phillips A (2010). Sea surface temperature variability: Patterns and mechanisms. Ann Rev Mater Sci.

[CR6] Deser C, Alexander M, Timlin MS (1996). Upper ocean thermal variations in the North Pacific during 1970-1991. J Clim.

[CR7] Deser C, Alexander M, Timlin MS (2003). Understanding the persistence of sea surface temperature anoMalies in midlatitudes. J Clim.

[CR8] Deser C, Phillips AS, Hurrell JW (2004). Pacific interdecadal climate variability: Linkages between the tropics and the north pacific during boreal winter since 1900. J Clim.

[CR9] Frankignoul C (1985). Sea surface temperature anoMalies, planetary waves, and air-sea feedback in the middle latitudes. Rev Geophys.

[CR10] Frankignoul C, Hasselmann K (1977). Stochastic climate models, part II: Application to sea-surface temperature anoMalies and thermocline variability. Tellus.

[CR11] Hasselmann K (1976). Stochastic climate models, part I: Theory. Tellus.

[CR12] Kang Y, Noh Y, Yeh S-W (2010) Processes that infuence the mixed layer deepening during winter in the north pacific. J Geophys Res, 115 (C12004)

[CR13] Kawabe M (2001). Interannual variations of sea level at the nansei islands and volume transport of the kuroshio due to wind changes. J Oceanogr.

[CR14] Kirtman BP, Pegion K, Kinter SM (2005). Internal atmospheric dynamics and tropical indo-pacific climate variability. J Atmos Sci.

[CR15] Kirtman BP, Schneider EK, Straus DM, Min D, Burgman R (2011). How weather impacts the forced climate response. Clim Dyn.

[CR16] Kirtman BP, Shukla J (2002). Interactive coupled ensemble: A new coupling strategy for CGCMs. Geophys Res Lett.

[CR17] Levitus S (1982) Climatological atlas of the world ocean. NOAA Prof. Paper, (13), 173 pp

[CR18] Luyten J, Pedlosky J, Stommel HM (1983). The ventilated thermocline. J Phys Oceanogr.

[CR19] Mantua NJ, Hare SR, Zhang Y, Wallace JM, Francis R (1997). A pacific interdecadal climate oscillation with impacts on salmon production. Bull Amer Meteor Soc.

[CR20] Narapusetty B, DelSole T, Tippett MK (2009). Optimal estimation of the climatological mean. J Clim.

[CR21] Narapusetty B, Stan C, Kirtman BP, Schopf PS, Marx L, Kinter JLIII (2012). The role of atmospheric internal variability on the tropical instability wave dynamics. J Geophys Res.

[CR22] Narapusetty B, Stan C, Kumar A (2014) Bias correction methods for decadal sea-surface temperature forecasts. Tellus A, (10.3402/tellusa.v66.23681), ISSN 1600–0870

[CR23] Newman M, Compo GP, Alexander M (2003). ENSO-Forced variability of the Pacific decadal oscillation. J Clim.

[CR24] Power S, Casey T, Folland C, Colman A, Mehta V (1999). Interdecadal modulation of the impact of ENSO on Australia. Clim Dyn.

[CR25] Qiu B (2003). Kuroshio extension variability and forcing of the Pacific decadal oscillations: Responses and potential feedback. J Phys Oceanogr.

[CR26] Saha S, et al. (2012) The NCEP Climate Forecast System veresion 2. J. Clim., (To be submitted)

[CR27] Saravanan R, McWilliams JC (1998). Advective ocean–atmosphere interaction: an analytical stochastic model with implications for decadal variability. J Clim.

[CR28] Schneider EK, Fan M (2007). Weather noise forcing of surface climate variability. J Atmos Sci.

[CR29] Stan C, Kirtman BP (2008). The influence of atmospheric noise and uncertainty in ocean initial conditions on the limit of the predictability in a coupled gcm. J Climate.

[CR30] Tomita T, Xie S-P, Nonaka M (2002). Estimates of surface and subsurface forcing for decadal sea surface temperature variability in the mid-latitude North Pacific. J Meteorol Soc Japan.

[CR31] Trenberth KE, Hurrell JW (1994). Decadal atmosphere-ocean variations in the Pacific. Clim Dyn.

[CR32] Yeh S-W, Kirtman BP (2004). The impact of internal atmospheric variability on the North Pacific SST variability. Clim Dyn.

[CR33] Yim BY, Noh Y, Yeh S-W (2012). Role of the ocean mixed layer processes in the response of the North Pacific winter sst and mld to global warming in cgcms. Clim Dyn.

